# Differential item functioning in neonatal behavioral neurological assessment in high-risk full-term infants in NICU based on a machine learning approach

**DOI:** 10.3389/fnins.2025.1681152

**Published:** 2025-11-18

**Authors:** Zhujiang Tan, Jinggang Wang, Jinwei Feng, Xuan Feng, Yi Huang, Kanglong Peng

**Affiliations:** Shenzhen Children’s Hospital, Shenzhen, Guangdong, China

**Keywords:** NBNA, Rasch model, differential item functioning, high-risk infant, machine learning

## Abstract

**Aim:**

This study adopted Rasch Analysis to evaluate the psychometric properties of the neonatal behavioral neurological assessment (NBNA) in high-risk full-term infants during their NICU stay.

**Methods:**

A total of 543 full-term infants (14.26 ± 7.02 days of age) were included in the study. We used the Rasch Model (RM) to assess the reliability and validity of the NBNA and GPCMlasso models to examine differential item functioning (DIF).

**Results:**

The samples responded to the NBNA according to the Rasch Model pattern. We found that the NBNA measures neurobehavior with one extra component regarding visual reactions. We found items that displayed disorder category functions in the NBNA. Conservatively, we found that the participants’ responses to the NBNA items were mostly dependent on the neurological developmental level, regardless of demographic traits.

**Conclusion:**

Our results support the applicability of the NBNA in depicting neurobehaviors in high-risk full-term infants in NICU. We found that high-risk infants could respond to NBNA items that were mostly dependent on the neural developmental level. The category functioning analysis revealed that the items provided inaccurate information owing to the disordered rating design.

## Introduction

Infants in the neonatal intensive care unit (NICU) are more likely to display abnormal developmental trajectories than their typically developing peers after discharge from the NICU, and more complicated problems may emerge as they grow older (Sucharew et al., 2012; Subedi et al., 2017). For example, infants who are discharged from the NICU may display hypotonia at the beginning and suboptimal motor function later, and some may present disorders in social communication when they enter kindergarten or even primary school (e.g., indifferent attitudes or insufficient social skills) (Sucharew et al., 2012). Longitudinal findings reveal that the developmental trajectory of these infants may vary: some may deviate downward from the average curve, and some may try to approach normal development, but most cases commonly have the same suboptimal developmental status (van Beek et al., 2021; Elbaum and Celimli-Aksoy, 2022). Studies have also revealed that infants discharged from the NICU are at a higher risk of poor academic achievement in regular education, even if they must obtain additional medical or educational resources to access the normal curriculum (Johnson et al., 2009; Hutchinson et al., 2013). For now, more efforts are needed to understand the neuromotor and associated psychological deficits secondary to complicated issue in infants discharged from NICU, and necessary monitoring is needed to prevent upcoming health threats including neurological damage (e.g., cerebral palsy), developmental delay, learning disorder, etc. (Bracewell and Marlow, 2002).

Previous studies have pointed out that timely early intervention programs are needed for those who were discharged from the NICU and presented unsatisfactory neurodevelopmental outcomes, and the results showed that these populations can reach age-appropriate skills in nearly all developmental aspects when they leave the programs (Elbaum and Celimli-Aksoy, 2022; Litt et al., 2018). These interventions can involve various developmental aspects and produce promising impacts on future academic functions in the early educational period, and require referral to these early intervention programs prior to timely hospital discharge (Hutchinson et al., 2013). To date, few factors have been found to determine whether infants discharged from the NICU should be referred to an early intervention program: birth weight, gestational age, NICU stay, medical comorbidity, and developmental status (Atkins et al., 2020; Atkins et al., 2017; Litt and Perrin, 2014). Despite increasing attention to NICU discharge follow-up, studies have found that a large number of eligible infants could miss the chance to receive necessary early intervention until they display significant delays compared to their typically developing peers (Atkins et al., 2017; Tang et al., 2012; Greene and Patra, 2016). Neurobehavioral assessments are clinical examinations designed for the preliminary period (e.g., the first few weeks) of neonates, which can provide useful information about their development. Several assessment tools have been used as predictors for early intervention enrolment in the first years of life for infants discharged from the NICU (Atkins et al., 2017; Rosinda et al., 2024; Spittle et al., 2008). To our knowledge, no assessment tool can portray the comprehensive contents of neurobehavioral profiles in infants discharged from the NICU (Spittle et al., 2008). Neurobehavior is broadly defined as the psychosocial and biological context of human experience (Salisbury et al., 2005). Neurobehavior consists of three dimensions: neurological items designed for active and passive tone, primitive reflexes, central neural system integrity, behavioral items, and stress/abstinence items (Salisbury et al., 2005; Malak et al., 2021; Als et al., 1977). The neonatal behavioral neurological assessment (NBNA) is established based on these dimensions to depict the following neurobehavioral components: behavior, passive tone, active tone, primary reflexes, and general status with excellent psychometric qualities (Bao et al., 1991; Bao et al., 1993; Zhang et al., 2021).

As timely identification of infants at risk for poorer prognosis is critical for NICU discharge, necessary measures are needed to obtain reliable information from infants in the NICU. The psychometric assumptions achieved by Classical Test Theory (CTT) depend heavily on the samples involved (Hambleton and Jones, 2005; Adedoyin et al., 2008; Peeters and Augustine, 2023). This method may lead to different psychometric results reported in different studies because the measurement accuracy was assumed to be invariant across all samples, regardless of personal characteristics (e.g., sex, gestational age, and delivery method), and the total score was utilized to estimate the measurement errors (Balasubramanian et al., 2024). Hence, to comprehensively explore the theoretical basis underlying the NBNA, we adopted the Rasch Model to illustrate the item-level psychometric properties in detail. Additionally, this study attempted to describe the magnitude of the measurement bias elicited by related variables in a clinical scenario. In addition, longitudinal follow-up was conducted to record the prognosis of infants who were discharged from the NICU in 2019. We aimed to build a prediction model for clinical practitioners to identify items that may require additional consideration for interpretation or for infants that may produce unexpected NBNA performance.

## Materials and methods

### Participants

Participants were recruited from hospital referral programs. Term infants with gestational age ≥38 weeks were admitted to the NICU were referred for early comprehensive evaluation using this program (Dolinskaya et al., 2023; Carlton et al., 2024). The referred infants would undergo an interdisciplinary assessment to determine their overall neurobehavioral status and join the follow-up program to obtain the necessary intervention. The comprehensive assessment routinely involved the administration of the NBNA and other standardized tools if needed. To achieve a reasonable sample size, this study categorized accompanying conditions according to International Classification of Disease (ICD). Prior to administration, all necessary consent was obtained from the legal guardians (s).

### Measure

The Neonatal Behavioral Neurological Assessment (NBNA) can serve as an observational rating tool to quantify neurobehaviors in infants admitted to the NICU. The NBNA consists of five testing components: behavior (six items), passive tone (four items), active tone (four items), primary reflexes (three items), and general status (three items). Each item is assigned a score from 0 to 2 where two denotes appropriate behavior and zero denotes behavior severely deviating from the normal criteria. The total score is the sum of all items, with scores < 35 denoting abnormal neurobehavior. The NBNA is performed by trained or licensed physicians or researchers.

### Data analysis

#### Analysis workflow

**Figure 1:**
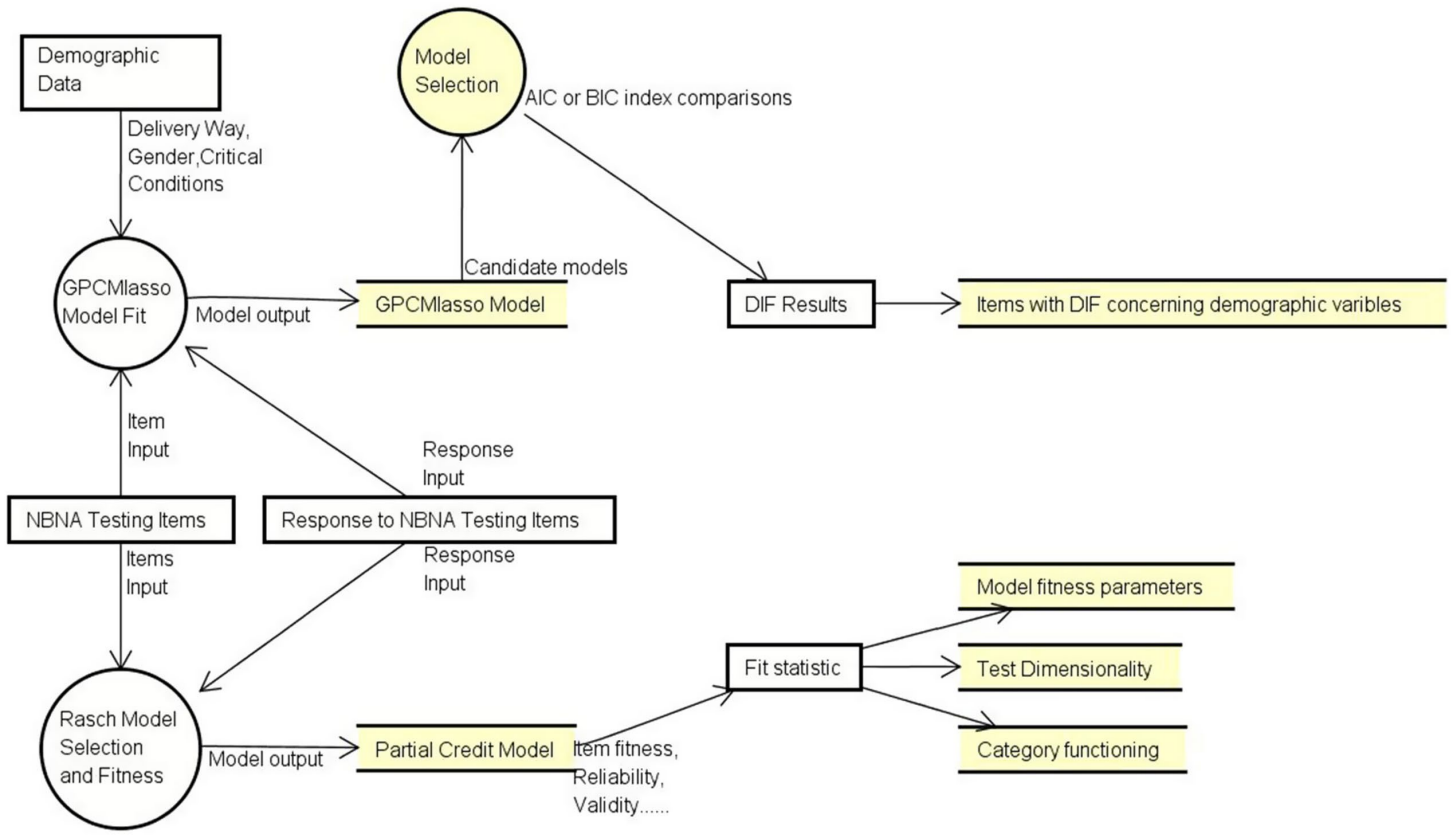
Analysis workflow in this study.

Figure 1 shows the overall analysis workflow that displays how our data is used and transfers in two different model fit procedures. Detailed descriptions are presented below. Two models were selected to simulate response data collected from infants at NICU. First, Rasch Model was utilized to test the response pattern and verify the psychometric quality of NBNA (e.g., reliability and validity) based on the fit statistic. Then, GPCMlasso Model was utilized to test DIF using demographic data (e.g., delivery way, gender). Detail description is presented below. Data was analyzed using WINSTEPS (Version 5.2.3.0, Copyright(c) 2022, John M. Linacre, website: http://www.winsteps.com) and R (RStudio, 2023.12.0 Build 369).

#### Rasch model

Rasch Model is widely known as an augment to Classical Testing Theory. The Rasch Model constructs an interval scale by converting the raw score to its natural logarithm. Instead of using the total score as the latent trait of the testing person, the Rasch Model adopted the item performance as the Sufficient Statistic to estimate the person’s ability (e.g., neurobehavioral status in this study) and item difficulty (e.g., the neural developmental milestone denoted by each item) independently. The possibility of successful/unsuccessful performance $x_{\upsilon \iota }$ is related to the difference between neurobehavioral status $\beta_{\upsilon }$ and developmental milestones $\delta_{\iota }$ , which allows us to estimate $\beta_{\upsilon }$ and $\delta_{\iota }$ independently from the available data. We can then simulate how these data fit the prediction model. The equations are as follows:

$$P\{x_{\upsilon \iota }=1|\beta_{\upsilon },\delta_{\iota }\}=\frac{\exp(\beta_{\upsilon }-\delta_{\iota })}{\left[1+\exp(\beta_{\upsilon }-\delta_{\iota })\right]}$$

The following psychometric properties were extracted from our simulation process: rater-related reliability, content-related reliability, internal consistency, and construct validity. The Rasch Model is widely utilized to test the psychometric properties of commonly used assessment tools including the Test of Infant Motor Development, Motor Proficiency 2nd Edition, Peabody Developmental Motor Scale, etc. (Wilson et al., 2011; Huang et al., 2018; Campbell et al., 2008; Dallmeijer et al., 2011; Chien and Bond, 2009).

In this study, the NBNA utilized a polytomous option design (e.g., 0, 1, 2); however, each item was rated based on its own criteria. For example, the criteria for scoring two points on Items 1 and 2 are different. Our study assumed that infants with a more optimal neural developmental status could display more behavioral patterns. Hence, we chose the Partial Credit Model (PCM) to examine the psychometric quality of the NBNA.

#### Item fitness

Item and person scores were transformed into natural logarithm units. The Rasch Model was then established based on the infants’ responses to the items. Item fitness reflects the prior assumptions of the Rasch Model. All Rasch measurements are built on the assumption that all items and infants should display acceptable fitness to the Rasch Model.

In this study, the infit mean square (MNSQ) and standardized Z (Zstd) were adopted to neutralize the impact of these unexpected performances by assigning corresponding weights to residuals. The MNSQ describes how much the participants’ responses may deviate from the model, and Zstd denotes how possible it is that the participants may generate unexpected responses. A reasonable deviation is defined by the infit mean square and Zstd, which fall within 0.75–1.33 and −2 to 2, respectively (Stolt et al., 2021; Sung et al., 2021; Ogawa et al., 2021).

#### Rater-rated and content-related reliability

Rater- and content-related reliability is defined as the measurement precision of the number of milestones distributed along the rating scale (e.g., analogical continuum). The person reliability index is used to measure the replicability of a person’s location on a continuum if another parallel set of items measuring the same construct is used. The item reliability index indicates the replicability of milestone locations along the continuum if these items are used by another group of people with the same demographic characteristics. Reasonable person and item reliability are supported with indexes beyond 0.8 (Stolt et al., 2021; Sung et al., 2021; Ogawa et al., 2021). A reasonable differential efficacy was established with a separation index greater than 2.0, indicating acceptable measurement precision.

#### Internal consistency

We used point measure correlation (PTMEASURE-CORR) to evaluate the relationship between the observations (e.g., actual performance) and measures (e.g., predicted performance). PTMEASURE CORR indicates the extent to which the items measure various aspects of the same latent trait. A negative correlation indicates that this item does not contribute to the traits that the measure intends to assess.

#### Construct validity

##### Category functioning

The NBNA used a 3-point rating scale (for example, 0, 1, 2). The Rasch Model defines the rating scale using ordering thresholds to separate different scores, and the threshold between different scores (e.g., threshold between “0” point and “1” points) is defined as the location on the ability continuum where there is a 50/50 possibility of scoring either of two adjacent points. Therefore, the NBNA contains two thresholds for each item, and these special ability landmarks are supposed to be aligned from left to right on this continuum. This means that individuals with a more optimal neural function status should obtain higher scores for each item. Hence, the interval between adjacent thresholds should cover a reasonable range (range from 1.4 to 5 is recommended) (Chang et al., 2014; Lu et al., 2013; Llamas-Ramos et al., 2018). In this study, we focused on the interval between the 0–1 threshold and 1–2 threshold. A well-designed category structure should assign an acceptable number of participants to an appropriate location on the ability continuum. Therefore, we expected that each category would be endorsed by at least 10 participants.

##### Unidimensionality

The Rasch Model utilizes point residuals between the actual and expected performance to conduct principal component analysis (PCA). The unidimensional structure is validated if NABA can explain over 40% of the residual variances, and the rest of the residual variances can be explained by random factors (eigenvalue less than 2.0). Additionally, the variance explained by the 1st contrast should be less than 15%, and the explained variance ratio of the measurement to the 1st contrast should be greater than 3:1 (Stolt et al., 2021; Sung et al., 2021; McCreary et al., 2013).

##### Differential item functioning

To address measurement bias, we used uniform DIF to evaluate the potential impact of related demographic variables. Various statistical methods have been introduced for DIF detection, and the Rasch Model is the most frequently used. However, a large sample was used in this study, which more easily elicited type 1 error. In addition, the Rasch Model can only focus on one variable and cannot eliminate the impact of other confounding factors. For example, the Rasch Model can only detect the DIF influence by gender on one trial, but these results may be contaminated by delivery way or other factors.

To fill the gap mentioned above, we used a machine learning method to establish a Rasch Model with a lasso penalty to identify the uniform DIF in the NBNA. The GPCMlasso R package was used to produce *λ* which denotes the magnitude of the influence produced by the covariances (e.g., delivery method, sex, critical conditions this study). Thus, uniform DIF is confirmed if this lasso coefficient is unequal to zero. In this study, a DIF analysis was conducted to test the influence of covariates, including delivery method, sex, and critical conditions.

Therefore, the calculation method can be written as follows:

$$\log(\frac{P(Y_{\mathrm{pi}}=r)}{P(Y_{\mathrm{pi}}=r-1)})=\beta_{i}[\theta_{p}+x_{p}^{T}-\delta_{\mathrm{ir}}-(\begin{array}{l} \gamma_{i1}*\text{Gender}+\gamma_{i2}*\\ \text{DeliveryWay}+\gamma_{i3}*\\ \text{CriticalConditions} \end{array})]$$

In this equation, $\gamma_{\text{in}}(n=1,2,3,4)$ denotes the influence of covariates on items i respectively. To modify the original DIF analysis methods (e.g., Welch’s t-test), the GPCMlasso package can test multiple covariates simultaneously and eliminate the potential multicollinearity that may exist among these variables. In this study, the Bayesian information criterion was adopted to screen for the optimal parameter *λ*.

### Sample consideration

To obtain 99% confidence that the item calibration (item difficulty measure) is within ±1/2 logit of its robust value and avoid type one errors. A sample size of over 250 is recommended to generate robust result in study using Rasch Model (Smith et al., 2008; Hagell and Westergren, 2016; Svanborg et al., 2022).

## Results

### Demographic data

A total of 543 term infants with normal birth weights were included in this study. Table 1 displays the overall demographic data for this sample. We managed to recruit a sample aged approximately 14.26 days. The sex ratio was 341/202 (boys/girls). Over 137 infants were delivered by cesarean section (25.2%), which is much higher than the 15% recommended by the WHO. In our study, 443 neonates were admitted to the NICU due to conditions that originated in the perinatal period, while 100 neonates were admitted due to other complicated postnatal issues.

**Table 1:** Participant demographic data.

Variables	Mean (SD)/Count (%)
Sample	543
Delivery way	
Natural delivery	406 (75.8%)
Cesarean	137 (25.2%)
Gender	
Female	202 (37.2%)
Male	341 (62.8%)
Age (days)	
Overall	14.26 (7.02)
Gestational age	
Overall (days)	274.35 (7.94)
Birth weight	
Overall (g)	3,167.33 (321.09)
Critical conditions	
ICD_00_Health conditions	3
ICD_01_Certain infectious or parasitic diseases	5
ICD_03_Diseases of the blood or blood-forming organs	1
ICD_05_Endocrine, nutritional or metabolic diseases	7
ICD_08_Diseases of the nervous system	22
ICD_11_Diseases of the circulatory system	8
ICD_12_Diseases of the respiratory system	5
ICD_13_Diseases of the digestive system	3
ICD_14_Diseases of the skin	1
ICD_15_Diseases of the musculoskeletal system or connective tissue	2
ICD_19_Certain conditions originating in the perinatal period	443
ICD_20_Developmental anomalies	15
ICD_21_Symptoms, signs or clinical findings, not elsewhere classified	21
ICD_22_Injury, poisoning or certain other consequences of external causes	6
ICD_25_Codes for special purposes	1

S.D., standard deviation.

### Person and item mapping and fit statistics

The item-person map showed that (see Supplementary material 1 for details) item 3 (“Reflection to GEGE-like sound) was the most difficult item (e.g., least observed behavior). This indicates that it is difficult for infants to respond to GEGE-like sounds. Item 6 (“Reflection to comfort”) was the most common behavior that could be easily observed in infants with different conditions. As the mean item frequency was set at 0 logit, item-person map shows that items in the NBNA are distributed symmetrically from 3 to −2 logit. This means that the NBNA can distinguish 83.34% of the behavior profiles in high-risk infants (e.g., 3 logit = 0.0474, −2 logit = 0.8808). The response pattern in this sample confirmed the hypothesis built by the Rasch Model (Table 2). This means that the following analysis results were established based on solid prior assumptions. The person reliability and separation index indicated that the NBNA is sufficiently efficient to distinguish infants with different neural developmental statuses. This means that the NBNA can capture all inter-person variations in neurobehavior instead of other irrelevant symptoms. A value of 0.74 means that 74% of the personal variants captured by the NBNA originated from interindividual differences and 26% were due to random errors. The item reliability and separation index revealed that the recruited sample size was reasonable for validating the item ranking on the scale continuum.

**Table 2:** Fit statistics summary of the NBNA.

Fit statistics		Total score	Count	Measure	Infit		Outfit		Real separation	Real reliability
					MNSQ	ZSTD	MNSQ	ZSTD		
Person	Mean	30	20	1.56	1.02	0.01	1.06	0.1	1.67	0.74
	S.D.	5.2	0	1.04	0.47	1.12	0.91	0.98		
Item	Mean	815.6	543	0	1.01	−0.12	1.07	−0.09	12.14	0.99
	S.D.	228.7	0	1.28	0.18	2.39	0.43	2.85		

S.D., standard deviation.

### Evaluation of item-fitness

In this study, we identified only nine items that violated the judge criteria (see Supplementary material 2). This means that these items may reflect some components that are not related to neural development, or these behaviors tend to happen randomly instead of patterns (e.g., slipping or accidentally presenting). Among them, we found only one item (item 15) that violated the MNSQ and Zstd criteria [for example, MNSQ (0.75–1.33), Zstd (−2–2)]. That means sucking activity may show up randomly instead of by appropriate stimulation (e.g., touching), and slipping here means it is possible that sucking activities will not show up even that suck reflex is already mastered by neonates. In general, outfit statistics denote the unstandardized residual between the expected and real values, and infit statistics represent the standardized residual that aims to eliminate the effect of erratic or robust patterns in this sample. MNSQ indicates the magnitude of the pattern deviation, while the Zstd indicates the possibility of an unexpected value.

In this study, most items displayed acceptable deviations from the model prediction, but the response pattern was too robust or erratic. For example, item 1 (“reflection to light) process normal MNSQ (for example 1.32) but unacceptable Zstd (for example 2.51), and this may propose that visual reflection to light was related to neurological status, but this behavior can also happen randomly due to other factors (e.g., environmental factors like sound, temperature). For example, item 15 (“sucking reflex) may be more suspected as random behaviors rather than neurological traits, and this behavior may not be a suitable behavior to calibrate the neurological status. In addition, all the items displayed positive PTMEASURE-CORR values, which means that all the items were related to the latent traits that the NBNA intended to examine.

### Category function

The category function analysis showed that four items (items 6, 18, 19, and 20) were not proportionally endorsed, especially item 18 (“Awake”). No response is endorsed in the “0” category for item 18. Three items did not display ordered threshold measures (items 1, 7, and 9), implying that the probability of individuals scoring specific points on these items may not be related to neurological status. Hence, we found that these items displayed abnormal threshold intervals. Other results also show nine items with abnormal threshold intervals, indicating that the original rating scale may be redundant. More details can be seen in Supplementary material 3.

### Assessment of unidimensionality

Principal component analysis revealed that the measurement variances explained by the NBNA were 51.3%. One contrast was found in the NBNA with an eigenvalue over 2 (for example, 2.38 in 1st contrast). This means that the NBNA measures more than just neurobehavior. The unexplained ratio of measured variances in the 1st contrast was 5.8%, and the raw explained variance ratio of the measure to the 1st contrast was 20.06/2.38 which was larger than 3/1.

To determine which of the NBNA items loaded into the residual factors, we set 0.4 as the cutoff value to define a meaningful factor loading (Martsolf et al., 2017; Maskey et al., 2018). Two items (e.g., item 4 vision speaking face, and item 5 vision red ball) displayed distinct dimensions of visual behavior.

### Differential items functioning

In the GPCMlasso equation, each group variable is encoded by the corresponding *λ*. To simplify the original equation, the GPCMlasso Model can be written as follows:

$$\log(\frac{P(Y_{\mathrm{pi}}=r)}{P(Y_{\mathrm{pi}}=r-1)})=[\theta_{p}-(\begin{array}{l} \beta_{i}+\gamma_{i1}*\text{DeliveryWay}+\\ \gamma_{i2}*\text{Gender}+\gamma_{i3}*\text{Conditions} \end{array})]$$

In this study, the GPCMlasso Model set the following characteristics in our sample as dummy codes: Delivery/nature delivery, Gender/girls, and Conditions/postnatal conditions were equal to 0 in this formulation (e.g., $\gamma_{i1}*\text{Cesarean}$ , $\gamma_{i2}*\text{boys}$ , $\gamma_{i3}*\text{perinatalConditions}$ ).

The Akaike Information Criterion (AIC) and Bayesian Information Criterion are widely used in model selection, and the AIC or BIC is defined as

AICORBIC(λ)=−2L(⋅)+df(λ)logn

where L(⋅) denotes the likelihood for the parameters estimated with tunning parameter *λ*, df(λ) denotes the total number of parameters estimated unequal to zero and n is the number of observations (Jafari et al., 2022). For a regular sequence of *λ* values, which was created by the GPCMlasso R package, the AIC or BIC was computed and sorted from largest to smallest values. The optimal λ is defined as which can lead to the smallest AIC or BIC value.

Table 3 represents the results of DIF detection using the GPCMlasso model in terms of the BIC and AIC criteria. Item DIF was examined between groups with different demographic traits. No Item DIF was found according to the BIC. Nine items displayed DIF for delivery way, gender, and critical conditions according to the AIC. For example, Table 3 shows that the Laplace coefficient *λ* for gender in item 7 (“Scarf sign”) is 0.3. For the same $\theta_{p}$ for children, the item difficulty for boys equals to $\beta_{i}$ , and $\beta_{i}+0.3$ for girls. This means that boys are more likely to maintain normal muscle tone than girls (e.g., the elbow cannot reach over the midline).

**Table 3:** The results of DIF analysis based on AIC method in the GPCMlasso model for variables in the NBNA.

Item	Content	Delivery/Natural	Gender/Girls	Condition/Postnatal
3	Auditory_GeGe	0.165	−0.254	0
4	Vision_Speeking_Face	−0.101	−0.266	0
7	Scarf_Sign	−0.02	0.303	0
8	Forearm_Rebound	0	0.042	0
10	Popliteal_Angle	0	0.362	0
13	Strech_Reaction	−0.048	0.011	0
14	Support_Reaction	0.03	0	0
16	Stepping	0.117	0	0
20	Mobility	0	0	0.04

Remark: Non-zero numbers under the group variable represent uniform DIF between subgroups.

An independent t-test was conducted to compare the subscale and total scores between the subgroups (Table 4). Our study only found significant differences between boys and girls in the behavior and passive tone subscales (*p* = 0.01 and 0, respectively).

**Table 4:** The NBNA scores comparison in DIF analysis regarding gender.

Components	Boys/N^1^	Girls/N	Levene’s test	*t*	*p* ^2^
	341	202			
Behavior	7.55 (1.96)	8 (2.02)	0.37	2.53	0.01
Passive tone^3^	6.74 (1.32)	6.34 (1.54)	0	−3.11	0.00
Active tone	5.2 (1.7)	5.08 (1.85)	0.27	−0.76	0.45
Primary reflex	5.06 (1.28)	5.02 (1.31)	0.79	−0.35	0.73
General status	5.55 (0.83)	5.46 (0.9)	0.09	−1.16	0.25
Total	30.11 (5.15)	29.91 (5.32)	0.28	−0.44	0.66

^1^N, sample size. ^2^*p*, *p* value. ^3^Unequal variances is assumed, and the Welch’t test and Mann–Whitney U test are conducted to produce both *p* values equal to 0.

## Discussion

Our study aimed to examine the psychometric properties of the NBNA using the Rasch Model and machine learning methods. This study aimed to describe the psychometric properties of the NBNA at the item level and address some limitations regarding potential assessment bias. Our findings confirmed that the overall response pattern in high-risk full-term infants with NICU stays can be well explained by the Rasch Model. In addition, the NBNA can explain over 51.3% of the measurement variance. An additional measurement component was identified for visual behavior. Our study proposes that sensory responses, especially visual interactions, can also reflect neurobehaviors in neonates. Our study also revealed some drawbacks regarding the rating design. Differences in characteristics traits can generate potential evaluation bias which can lead to variant psychometric properties across different subsample diagnoses as high-risk infants.

### Measurement properties of the NBNA items

The overall response pattern of high-risk infants shows that individuals with a more optimal neural developmental status can perform more neurobehaviors in the NBNA. Our study concurs with previous research that intraindividual variability can be observed in infants at a few days (de Weerth et al., 1999; Robertson, 1993). This is the reason why the measurement component may display a high probability of an unexpected response, and these variations are limited within an acceptable range.

In the NBNA, three items were not proportionally endorsed. Among them, we found that no infants remained unconscious, which means that most infants can remain in normal sleep–wake cycles (e.g., 2 points in item 18) or drowsiness (e.g., 1 point in item 18). This finding is in line with previous findings that the arousal state is important during neurological or neurobehavioral examinations (Brown and Spittle, 2014). The unreasonable category function may cause the following problems. First, unnecessary time is needed to judge which score is appropriate due to redundant scoring design (e.g., zero is unnecessary in item 18). Second, inaccurate information may be given due to inappropriate scoring curve. For example, we found that the cutoff ability scale between zero and one point is 0.26 while it is −0.26 between one and two points. Third, unreasonable intervals may be created. The interval between ascending point along the ability continuum may be too narrow to distinguish participants with different neurological status.

### Measurement unidimensionality

For dimensionality analysis, the NBNA explained 51.3% of the measurement variance. The analysis also revealed an additional meaning component within the NBNA: visual behavior. Neonates in the NICU experience both sensory overload and deprivation (Lott, 1989). In our study, two visual reaction items constitute one extra meaning dimension apart from neurobehavior. One study found that temperament profiles in neonates may affect sensory reactions in early infancy (DeSantis et al., 2011). Another study found that arousal status also has a remarkable impact on environmental interaction regarding external stimuli (e.g., light, sound) (Roy et al., 2004). Our study supports the notion that sensory responses or engagement can be affected by various factors apart from neurological status (DeSantis et al., 2011; Roy et al., 2004). Hence, more studies are needed to discuss whether the visual components should be extracted from the original behavior section.

### Differential items functioning

To date, our study is the first to focus on DIF detection in the NBNA. In addition, our study is the latest study using machine learning methods for DIF analysis. Two model selection methods were used in our study: AIC and BIC (Vrieze, 2012). BIC was selected as the criterion to produce more conservative parameters than AIC (Schauberger and Mair, 2020). The BIC assumes that as the sample size increases, the number of parameters in the true model is finite. This means that as the sample size grows sufficiently large, the true model will be selected. According to the BIC method, our study revealed that no items in the NBNA displayed different DIF in different groupings. This may imply that these populations may respond to NBNA items depending on their neural developmental level only. The identification of items with DIF using the AIC criteria emphasizes that specific considerations regarding individual characteristics if more participants were involved (e.g., gender and critical conditions in this study) are needed to interpret the NBNA score at the item level.

For gender DIF, all NBNA items displayed an outstanding ability to capture neural behaviors in children equally. However, previous findings have revealed that sex can be a significant variable in predicting neuromotor behavior in infants (Gabis et al., 2021; Ramoğlu et al., 2016; Samsom et al., 2002; Samsom et al., 2002). Study found that neural structure differences can be noticed between boys and girls at term-equivalent age (Liu et al., 2011; van Kooij et al., 2011). To investigate whether these structural differences were related to sex differences in neurobehavior, various findings offer researchers unique opportunities to observe neural behavior differences in neonates at a time when environmental impact was still minimal. In our study, we found that boys were superior to girls in passive muscle tone, and we identified four items that were advantageous to boys at the item level, while girls performed better in two items. In line with previous finding, there are more similarities than differences between boys and girls in the neonatal period in terms of neurobehavior (Samsom et al., 2002; Samsom et al., 2002).

In this study, we adopted the GPCMlasso model to examine the potential impact of multiple related demographic variables simultaneously. This method can address the statistical limitations mentioned in previous studies (Boatella-Costa et al., 2007; Lundqvist and Sabel, 2000). Previous studies have shown that neurobehavioral differences between sexes can be ambiguous when using classical testing theory (e.g., t-test in this study or cited studies). The GPCMlasso model using the AIC method can address these ambiguous relationships between sex and neurobehaviors if the samples used in our study are large enough and the parameters used in the formulation are sufficient. Hence, we conclude that gender DIF is not true in our current sample (e.g., neonates who are born with normal born weight and gestational age). And we are not sure whether this is also true in other samples such as neonates who are born with low born weight or shorter gestational age. More rigorous studies are needed.

In summary, the GPCMlasso model can explain the current data set used in our study and may not remain true for other data sets or may be altered if any one of the sample demographic characteristics are changed. Nevertheless, model selection is not hypothesis tests and does not draw determined conclusions as to whether candidate models are true or false. Instead, it only explores and ranks all the candidate models. For complex human conditions, especially neonates’ status, we cannot expect that statistical methods alone can perfectly simulate such complex situations. But, model selections can provide reasonable frameworks to included information, such as gender in this study, more flexibly (Li and Nyholt, 2001).

### Implications for clinical practice

Our results provide preliminary evidence to support the conclusion that the NBNA contains a reasonable measurement structure aimed at capturing neurobehaviors in infants. Moreover, our findings revealed that high-risk infants responded to NBNA items mostly depending on their neural developmental status. But this may not be true if the minority in our sample grows larger (e.g., neonates delivered by cesarean or born preterm). We also found that some shortcomings may jeopardize the psychometric properties of the NBNA. For example, when neonate failed in item 15 (“sucking reflex”), more consideration is needed to judge if suck reflex is mastered by neonates since they tended to slip or perform this item randomly. This reminds clinicians to interpret the scoring points in the NBNA with additional caution. In particular, clinicians need to make decisions using scores that approach the judgment threshold (e.g., 35 points in this study).

### Study limitations

From a statistical perspective, our study failed to recruit an equivalent subsample size in each subgroup (e.g., preterm or term). In addition, the GPCMlasso model is only useful for detecting uniform DIF; hence, we cannot find other items with non-uniform DIF. This means that if an item displays inconsistent DIF in subsamples, we would be able to find it. For example, infants with lower birth weights (e.g., Extremely low birth weight) would fail to display more optimal behaviors in items, but infants with heavy birth weights (e.g., low birth weight) can display better behaviors. The DIF pattern cannot maintain stability along the entire ability continuum (e.g., developmental level in this study). It is more complicated to use a penalized likelihood function to determine items with non-uniform items, even though it may be theoretically feasible.

## Conclusion

Our results support the applicability of the NBNA in depicting neurobehavior in high-risk infants with NICU stays. We found that high-risk infants could respond to NBNA items that were mostly dependent on the neural developmental level. Our study did not find any demographic bias according to the BIC method. In addition, our findings concur with previous assumptions that vision reaction is essential for depicting neurobehavior in high-risk infants.

## Data Availability

The datasets presented in this article are not readily available because for paticipants’s privacy right, data is not available. Requests to access the datasets should be directed to Kanglong Peng 18096723g@connect.polyu.hk.
