# Supplementary figures and images for: Differential item functioning in neonatal behavioral neurological assessment in high-risk full-term infants in NICU based on a machine learning approach
Source: Front Neurosci. 2025 Nov 18;19:1681152. doi: 10.3389/fnins.2025.1681152 (PMC12669220; doi:10.3389/fnins.2025.1681152)

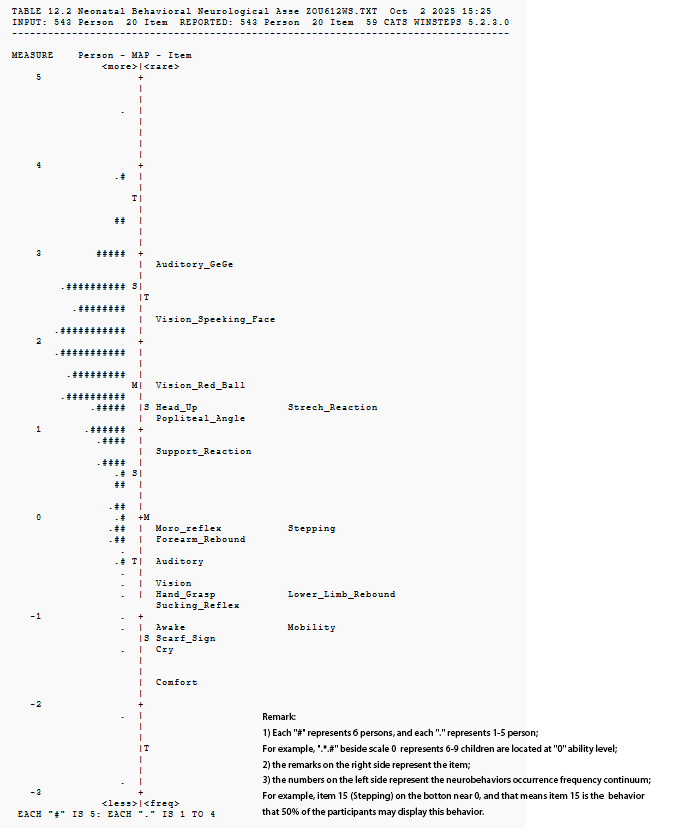

Supplement: Supplementary file 1 [file Image_1.jpg]
